# Influence of vascular geometry on local hemodynamic parameters: phantom and small rodent study

**DOI:** 10.1186/s12938-018-0458-8

**Published:** 2018-03-02

**Authors:** Lili Niu, Xiliang Zhu, Min Pan, Abbott Derek, Lisheng Xu, Long Meng, Hairong Zheng

**Affiliations:** 1 Paul C. Lauterbur Research Center for Biomedical Imaging, Institute of Biomedical and Health Engineering, Shenzhen Institutes of Advanced Technology, Chinese Academy of Sciences, 1068 Xueyuan Ave., Nanshan District, Shenzhen, 518055 People’s Republic of China; 2 Department of Cardiovascular Surgery, Henan Province People’s Hospital, Fuwai Central China Cardiovascular Hospital, Zhengzhou University People’s Hospital and Medical School of Henan University, Zhengzhou, 450000 Henan Province People’s Republic of China; 3 Sino-Dutch Biomedical and Information Engineering School, Northeastern University, 195 Chuangxin Ave., Hunnan District, Shenyang, 110819 People’s Republic of China; 4 Key Laboratory of Medical Image Computing, Ministry of Education, Northeastern University, Shenyang, China; 5 Guangzhou University of Chinese Medicine, Guangzhou, People’s Republic of China; 6 Centre for Biomedical Engineering, School of Electrical and Electronic Engineering, University of Adelaide, Adelaide, Australia

**Keywords:** Ultrasound imaging, Hemodynamics, Wall shear stress, Carotid bifurcation, Atherosclerosis

## Abstract

**Background:**

Many studies have demonstrated that the geometry of the carotid bifurcation enables prediction of blood flow variation associated with atherosclerotic plaque formation. The phase angle between the arterial wall circumferential strain and its instantaneous wall shear stress is known as stress phase angle (SPA). This parameter is used to evaluate hemodynamic factors of atherogenesis. Note that SPA can be numerically computed for the purpose of locating atherosclerosis in different artery geometries. However, there is no experimental data to verify its role in the location of atherosclerosis in different artery geometries. In this study, we use an ultrasonic biomechanical method to experimentally evaluate the role of SPA for locating atherosclerosis in carotid bifurcation.

**Results:**

For carotid anthropomorphic vascular phantom experiments, the SPAs of common carotid arteries (CCAs), external carotid arteries (ECAs) and internal carotid arteries (ICAs) are − 148.53 ± 6.92°, − 153.95 ± 5.11°, and − 238.69 ± 1.72°, respectively. The corresponding SPAs are − 173.47 ± 0.065°, − 115.57 ± 4.83° and − 233.9 ± 8.12° for the polyvinyl alcohol (PVA-c) phantoms. In vivo mouse experiments indicated that the wall shear stress and circumferential strain were out of phase in the ICAs (− 280.08 ± 13.12°) to a greater extent as compared to CCAs (− 141.97 ± 8.03°) and ECAs (− 170.07 ± 9.24°).

**Conclusions:**

The results suggested that SPA may be a useful indicator to locate the atherosclerosis position in carotid bifurcation.

## Background

Atherosclerotic plaques are localized and they are most likely to occur at arterial bifurcations and bends [1, 2]. Many studies have demonstrated that the mechanical forces exposed to arterial endothelial cells play an important role in the development and progression of atherosclerosis [3–6]. These forces mainly include wall shear stress (WSS) as a result of blood flow and the circumferential strain (CS) caused by periodical wall motion and pulsatile pressure. Since these forces are primarily determined by the arterial geometry, some regions of the artery may have an increased risk of developing atherosclerosis [7], particularly for arterial bifurcations. Recent research has shown that the plaque formation is most obvious at the outer wall of the carotid sinus where the flow separates [8–10]. Furthermore, arterial remodeling and the development of atherosclerotic disease need to be evaluated by the circumferential wall stress, an essential parameter for determining atherogenesis [7, 11–13]. Therefore, the CS and WSS can be used as indicators for carotid bifurcation atherosclerosis. The temporal phase angle between CS and WSS, which we have termed as the stress phase angle (SPA), determines the influencing effect of arterial wall shear stress and blood shear stress [13, 14]. Note that SPA has been proven as a potential important parameter due to its role in the pathophysiology of vascular disorders [15]. Furthermore, several numerical investigations on SPA have been carried out to locate the positions of atherosclerosis in different geometric arteries. Carotid bifurcation modelling using fluid–structure interaction shows that large negative SPA values are located at the atherosclerotic plaques attached to the outer wall of the carotid sinus [16]. In addition, some studies also demonstrated that SPA had significant value for predicting stenosis severity of artery [17, 18]. However, the role of SPA for locating atherosclerosis in different geometric arteries has not been verified experimentally yet.

Recently, an ultrasonic biomechanical (UBM) method was established to characterize the displacement and flow pattern of arterial wall simultaneously [19–22]. This method is employed to quantitatively determine and evaluate the function of SPA in predicting early atherosclerosis based on an experimental platform. Results have shown that highly negative SPA correlates to arterial wall stiffening [23]. The UBM method has been utilized in this experimental study to analyze the role of SPA for location of atherosclerosis in different geometric arteries. Firstly, the changes of SPA in different locations of the carotid artery are studied by UBM. High levels of negative SPA could be found in the internal carotid artery (ICA) as compared to the common carotid artery (CCA) and external carotid artery (ECA). Secondly, the results are verified by in vivo mouse experiments. Experimental results demonstrated that a greater negative SPA value was indicative of arterial disease risk.

## Methods

### Experimental setup

The UBM method is capable of measuring the displacement of vessel wall and blood flow simultaneously, which has been demonstrated in [24]. Further detail on this method for calculating the SPA is given in [23], and this method has been used to analyze SPA temporal distribution with arterial stiffening in an experimental setup. The data file acquired contains frames over several cardiac cycles. Contrast-enhanced images are computed between consecutive frames using the UBM method. Firstly, ROIs are selected including the vessel wall and lumen. Each ROI is divided into a grid of small sections known as interrogation windows. Then, the UBM method is used to calculate the displacement of each interrogation window. This process is repeated at each interrogation window within the image, resulting in a map of displacement vectors to describe the arterial wall movement and flow. The CS can be obtained from the displacement of vessel wall, and WSS can be determined based on gradient of the flow velocities. Therefore, the SPA can be acquired by the temporal phase angle between CS and WSS.

**Fig. 1:**
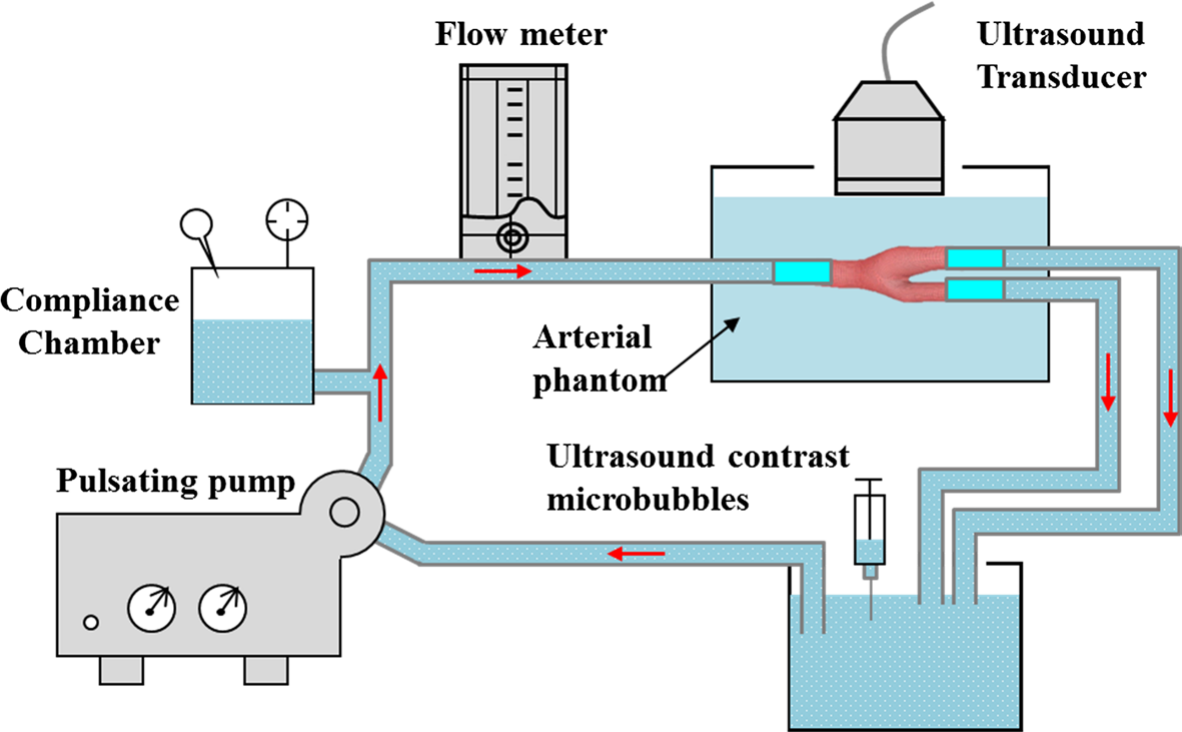
Experimental set-up was established to calculate the circumferential strain and wall shear stress of carotid anthropomorphic vascular phantoms and the polyvinyl alcohol (PVA-c) phantoms using the ultrasonic biomechanics method

A closed-loop compression system can be used to pressurize an arterial phantom (Fig. 1) and an ultrasound imaging system to acquire the image sequence. Pulsatile flow, mimicking the ventricular action of the heart, was generated by a Harvard Apparatus pulsatile pump (Model 55-3305; Harvard Apparatus, Holliston, MA, USA). Ultrasound contrast microbubbles containing cores of octafluoropropane (C_3_F_8_) gas surrounded by a phospholipid shell are developed. A 0.025 ml volume of microbubbles was injected into the upstream chamber and mixed to ensure uniform bubble concentration during each measurement. Then, we performed microbubble seeding as described in the previous section [25]. The transducer was placed perpendicular to the flow direction to obtain the ultrasound contrast images.

### Phantom experiments

**Fig. 2:**
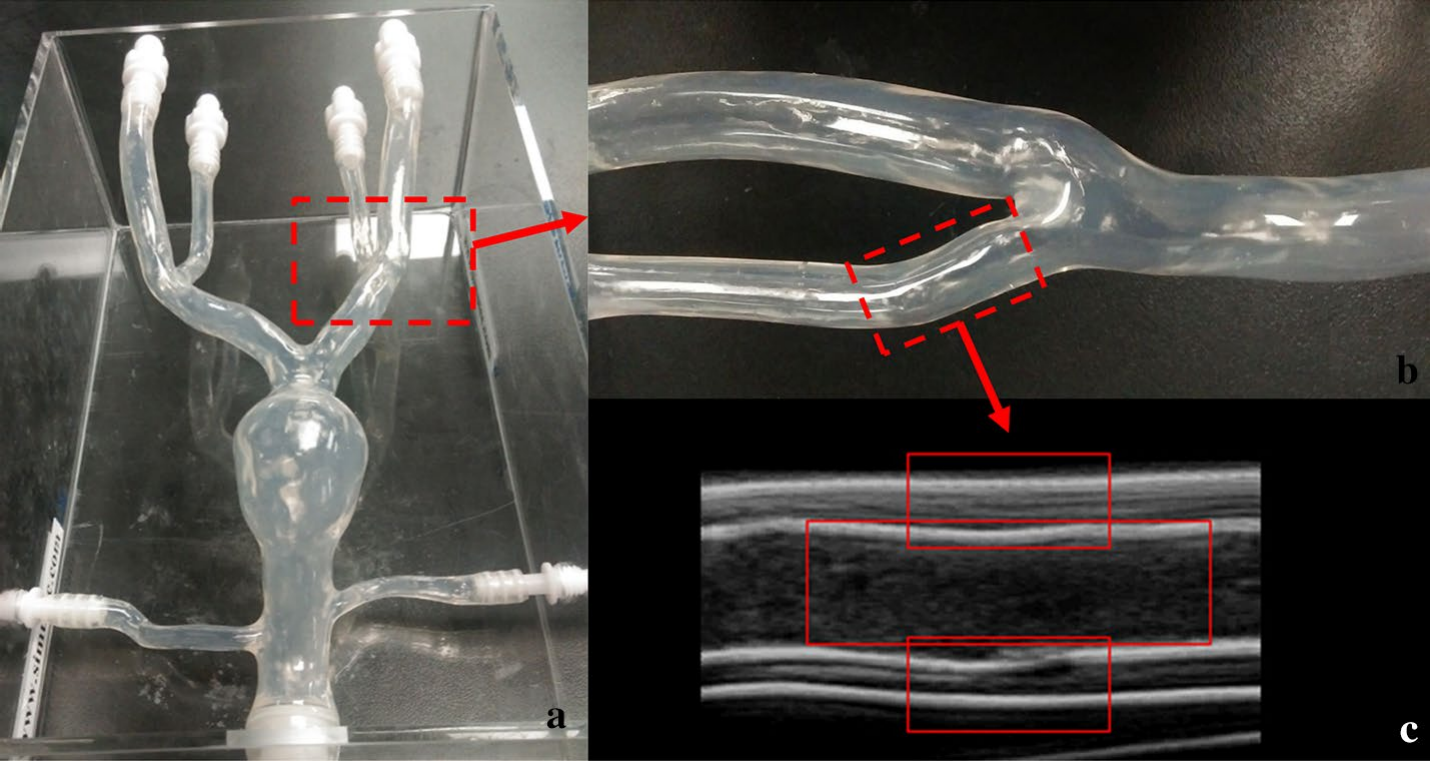
**a** A carotid anthropomorphic vascular phantom representing a normal carotid bifurcation; **b** the enlarged image of carotid bifurcation; **c** the ultrasound contrast image of external carotid artery

A carotid anthropomorphic vascular phantom manufactured by Shelley Medical Imaging Technologies (London, ON, Canada) for mimicking a normal carotid bifurcation was used to study the change of SPA at different locations of the carotid bifurcation (Fig. 2). An open-architecture ultrasound system (Sonix RP, Vancouver, Canada) with a 10 MHz linear array transducer was used to image the vascular phantom in the longitudinal section. The frame rate was 125 Hz and the field of view was 40 mm (depth) × 20.8 mm (width).

**Fig. 3:**
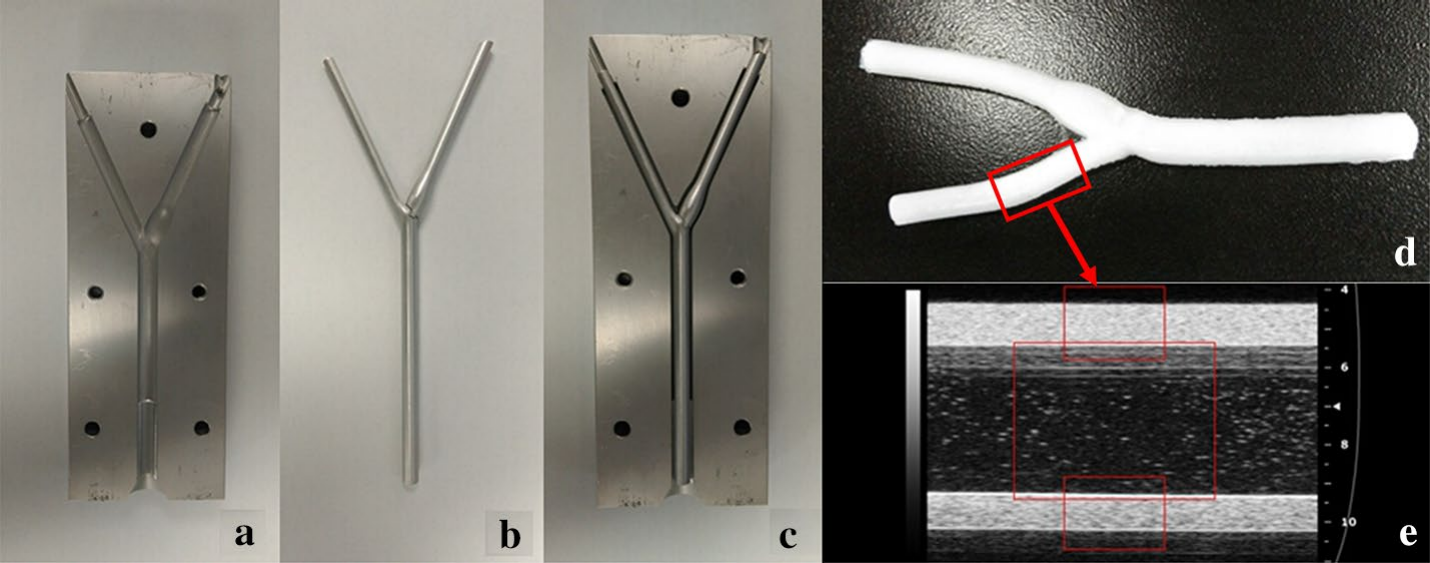
The carotid bifurcation mold consisting of **a** a polished stainless-steel shell, and **b** a bifurcated inner rod (6 mm CCA diameter, 4.5 mm ICA diameter, 4 mm ECA diameter). **c** All the components were assembled together, and the PVA solution was injected into the gap. **d** The resulting carotid bifurcation vessel phantom. **e** The ultrasound contrast image of external carotid artery

In addition, the arterial phantom was made of PVA cryogel (PVA-c). The PVA aqueous solution was filled into a steel mold, and then frozen and subsequently thawed to form a cryogel with rubber-like properties [26]. The composition (by weight) of the solution was 10% PVA powder, 87% deionized water, and 3% scattering particles composed of sigmacell cellulose with diameter of 20 μm (Sigma-Aldrich, USA). The mold consisted of two polished stainless-steel shells, a bifurcate inner rod (6 mm CCA diameter, 4.5 mm ICA diameter, 4 mm ECA diameter), and two hexagonal screws, as shown in Fig. 3a, b. The solution was injected into the gap between the rod and the shells (Fig. 3c). The whole mold underwent a number of freeze–thaw cycles to generate higher stiffness. Each freeze–thaw cycle was composed of 12-h freezing at − 20 °C and 12-h thawing at 20 °C. The optical photo and the ultrasound image of the carotid phantom are shown in Fig. 3e, f. The stiffness of the PVA phantom was also tested on an electronic universal material testing machine (CMT6104; New Sans Machinery Co., Ltd., Shenzhen, Guangdong, China). The phantom with three freeze–thaw cycles (Young’s modulus, 162.45 kPa) was used in flow experiments. The carotid phantom was imaged using a high frequency small-animal imaging system with a 40-MHz linear transducer (Visualsonics). The image acquisition frame rate was 125 Hz and the number of the image sequence was 800.

### In vivo mouse carotid artery experiments

The change of SPA in carotid bifurcation was further investigated in vivo. Our animal experiments were conducted in strict accordance to protocols that are approved by the Institutional Animal Care and Use Committee of Shenzhen Institutes of Advanced Technology, Chinese Academy of Sciences. During the injection and image acquisition process, mice were anesthetized with 1.0–2.0% isoflurane in oxygen delivered at a flow rate of 1.0 min/l and monitored with electrocardiogram. Using a rectal temperature probe, body temperature was carefully maintained between 36.7 and 37.3 °C throughout the study. Ultrasound contrast agents composed of octafluoropropane (C_3_F_8_) gas encapsulated by a phospholipid shell fabricated in house were injected through the tail vein into each mouse with a 2.0 × 10^5^ microbubbles/ml concentration. Ultrasound biomicroscopy (Vevo 2100, Visualsonics, Toronto, Canada) with a transducer frequency of 40 MHz was used for vascular imaging in anesthetized mice. The frame rate was 200 Hz.

### Statistical analyses

The analysis of covariance (ANOVA) was used to examine the role of SPA for locating atherosclerosis in carotid bifurcation. A p value less than 0.05 was accepted as indicating statistical significance. All statistical analyses were performed using the Statistical Package for Social Sciences statistical software package, version 17.0 (SPSS Inc.).

## Results

### Carotid anthropomorphic vascular phantom experiments

**Fig. 4:**
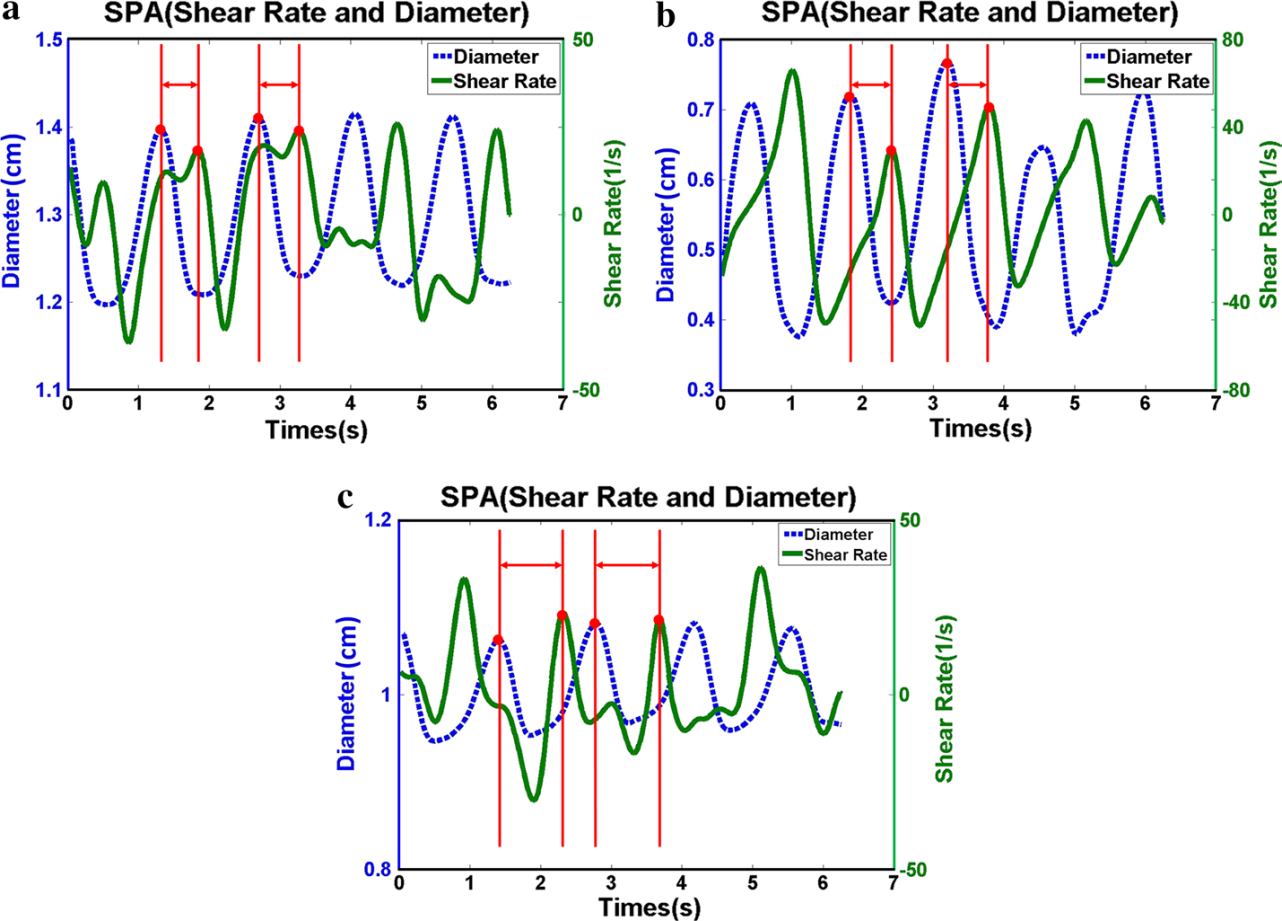
The distribution of arterial diameter and wall shear rate for carotid anthropomorphic vascular phantoms in the common carotid arteries (**a**), external carotid arteries (**b**), and internal carotid arteries (**c**)

The UBM method was used to measure the measure hemodynamic parameters in the carotid anthropomorphic vascular phantom experiments. The distribution of arterial diameter and wall shear rate for vascular phantom in the CCA (a), ECA (b) and ICA (c) was shown in Fig. 4. It is worthwhile noting that the diameter and WSR curves exhibit a periodical variation and phase shift. The SPA values located at the CCA, ECA and ICA are − 148.53 ± 6.92°, − 153.95 ± 5.11°, and − 238.69 ± 1.72°, respectively. It can be observed that SPA is more negative for the ICA where the atherosclerotic plaque develops.

### PVA-c phantom experiments

**Fig. 5:**
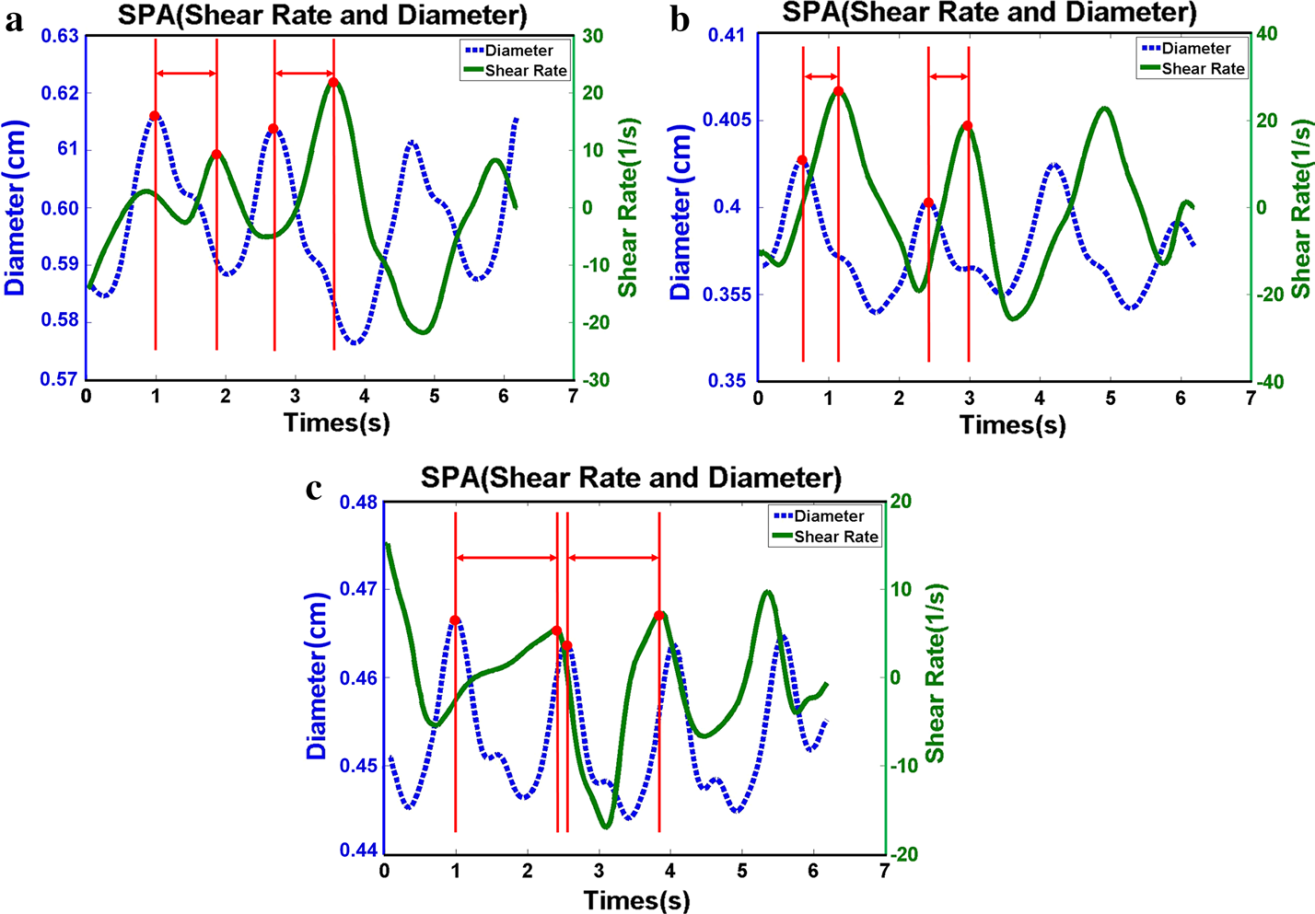
The distribution of arterial diameter and wall shear rate for PVA-c phantom in the common carotid arteries (**a**), external carotid arteries (**b**), and internal carotid arteries (**c**)

The PVA phantoms are performed to verify the accuracy of the results in carotid anthropomorphic vascular phantom. The temporal diameter and WSR curves of the CCA, ECA and ICA in the PVA phantom are shown in Fig. 5a–c, respectively. The diameter and WSR curves show a periodical variation and phase shift. Note that the SPA, WSR and the strain at different locations of the PVA phantoms are shown in Table 1. There is a 35% increment in negative SPA, a 27% reduction in WSR, and a 26% reduction in strain pertaining to the ICA in comparison to that of the CCA.

**Table 1:** Parameters of the stress phase angle (SPA), wall shear rate (WSR) and arterial strain in different locations of PVA-c phantoms

Location	SPA (^o^)	WSR (1/s)	Strain (%)
CCAs	− 173.47 ± 0.065	21.94 ± 0.125	4.31 ± 0.33
ECAs	− 115.57 ± 4.83	26.13 ± 0.51	2.35 ± 0.055
ICAs	− 233.9 ± 8.12	16.06 ± 0.92	3.18 ± 0.05

*CCAs* common carotid arteries; *ECAs* external carotid arteries; *ICAs* internal carotid arteries

### In vivo mouse carotid artery experiments

**Fig. 6:**
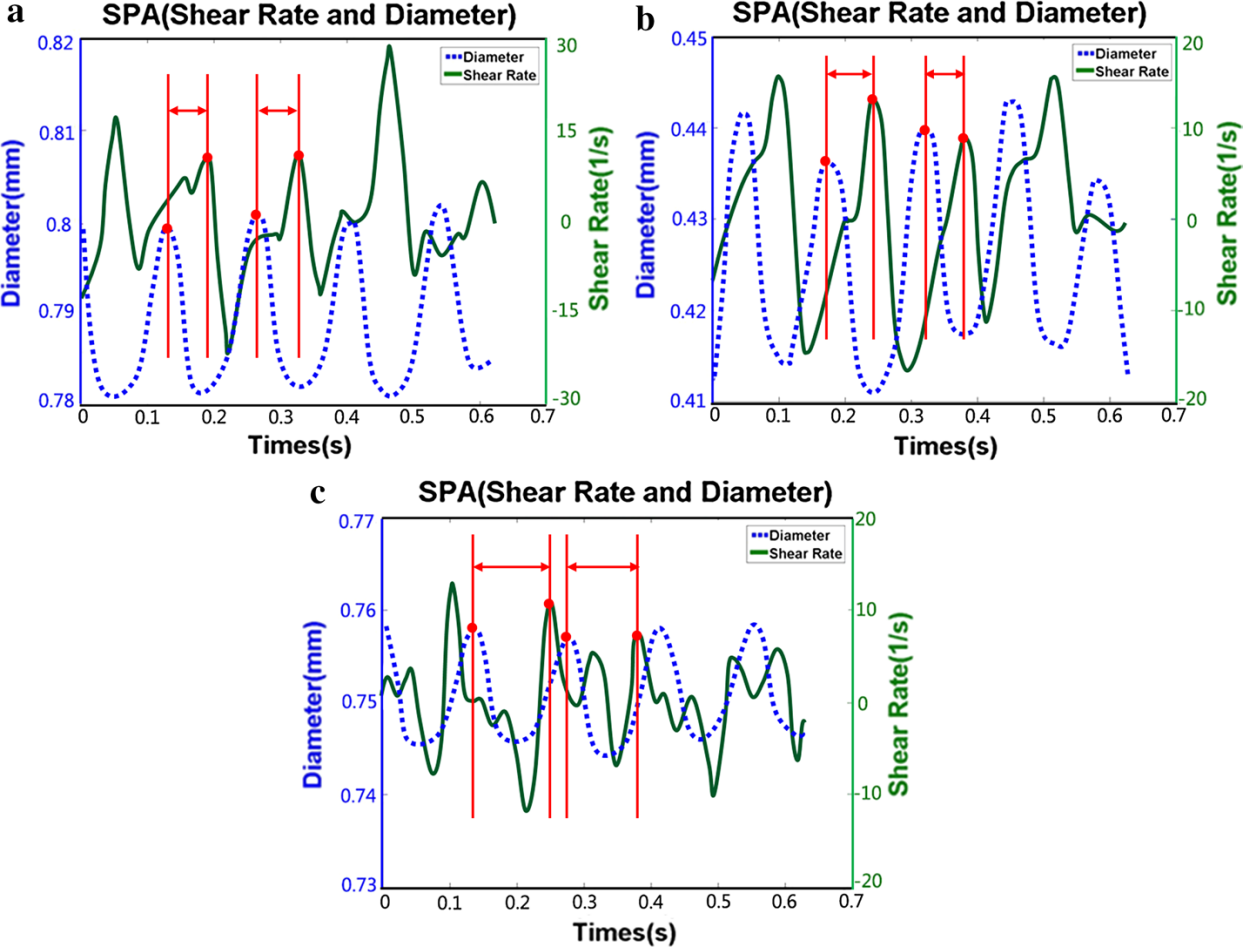
The distribution of arterial diameter and wall shear rate in mice common carotid arteries (**a**), external carotid arteries (**b**), and internal carotid arteries (**c**)

The UBM method is utilized in determining the flow velocity distributions and the arterial diameter changes of the CCAs, ECAs and ICAs present in mice. The change of diameter and WSR profiles with time is depicted in Fig. 6. It is notable that there are periodic patterns between the diameter and WSR and the phase shift at various times. The SPA, WSR and the strain at various locations of mice are shown in Table 2. Here, more negative SPA is achieved in the ICAs (− 280.08 ± 13.12°) compared with CCAs (− 141.97 ± 8.03°) and ECAs (− 170.07 ± 9.24°), while correspondingly, WSR and strain are lower in the ICAs. A comparison of different locations was made by analysis of variance. Significant differences were found among locations for SPA (*p* = 0.0001), WSR (*p* = 0.045) and strain (*p* = 0.009).

**Table 2:** Values of stress phase angle, wall shear rate and arterial strain in specific locations of carotid arteries in mice

Location	SPA (^o^)	WSR (1/s)	Strain (%)
CCAs	− 141.97 ± 8.03	28.45 ± 3.82	19.07 ± 2.15
ECAs	− 170.07 ± 9.24	21.68 ± 2.99	13.62 ± 2.40
ICAs	− 280.08 ± 13.12	19.82 ± 3.23	11.68 ± 1.15
F	148.84	5.45	11.32
p	0.0001	0.045	0.009

The abbreviations are as in Table 1

## Discussions

This study showed that SPA was highly dependent on geometries and it might be an essential predictor in early atherosclerosis. Note that CS and WSS were not synchronous in the ICA as verified by in vitro carotid anthropomorphic vascular phantoms. Moreover, the SPA is large and negative for the ICA where atherosclerotic plaques located. The results were further demonstrated by PVA-c phantom experiments and in vivo mouse carotid artery experiments. Previous studies have indicated that SPA appears to be negative at higher degrees in positions that depict flow separation having low shear stress in comparison to the non-separated positions that have high shear stress. Lee and Tarbell performed experimental studies in a compliant aortic bifurcation model and found that the SPA was − 40° on the flow divider (high shear) and − 100° on the outer wall (low shear) [27]. Tada and Tarbell showed that the SPA became highly negative (approaching − 180°) along the entire length of the carotid sinus outer wall [16]. The results are consistent with our measurements and computations of the SPA in the carotid bifurcation and large negative SPA in precisely those regions of arteries where atherosclerotic disease usually develops.

Many studies have shown that the development of atherosclerosis in the naturally bulbic ICA may be due to the local hemodynamic conditions, such as reduced and oscillating WSS [7, 28, 29]. Although cardiovascular risk factors typically cause the thickening and stiffening of the CCA wall, the development of atherosclerosis at the naturally dilated ICA bulb is mainly related to the bifurcation geometry [2]. Our findings indicate that the WSR is found to be significantly reduced in the ICA, as shown in Tables 1, 2. Our results offer further evidence that the area of atherosclerotic plaque development has a higher tendency for low WSS values and indicates that the geometry of the artery may contribute to subclinical atherosclerosis. The regions at relatively low WSS are also characterized by more negative values of SPA in the phantom and mice experiments as described in this study. Therefore, the geometry and unique hemodynamic characteristics (SPA = − 238.69 ± 1.72° for vascular phantom; SPA = − 233.9 ± 8.12° for PVA-c phantom; SPA = − 280.08 ± 13.12° for mice) of ICAs may contribute to predicting high-risk atherosclerosis regions in these vessels.

The SPA combines the information of both WSS, which was resulted from blood flow, and the CS caused by periodical wall motion and pulsatile pressure. The results suggest that flow separation and low WSS areas comprise the most negative SPA (Figs. 4, 5, 6), which is consistent with previous studies [2, 29, 30]. Biological studies have demonstrated that negative SPA can inhibit anti-atherogenic gene expression and release, but at the same time, they also increase pro-atherogenic gene expression and metabolite release [31, 32]. These results proved that ECs driven by hemodynamics and wall mechanics can potentially cause a pro-atherogenic effect on regions of the local circulation.

There were several limitations of this study: (1) The ultrasound data for CCA, ICA and ECA were collected independently at different initial phases. The SPA is independent of the initial phase, thus the results are unaffected. (2) Previous studies addressed that the acoustic properties of the experimental phantoms (artificial tissue, vessels, and blood) should match to those of actual human tissue [33]. Future studies will take into account these effects. (3) Given the small number of animals, our findings must be regarded as preliminary. Future research with larger sample size will be required to further verify these findings [34].

## Conclusion

The determination and analysis of SPA is proposed for locating the positions of atherosclerosis in different geometric arteries based on the UBM method. The SPA of CCA, ECA and ICA is − 148.53 ± 6.92°, − 153.95 ± 5.11°, and − 238.69 ± 1.72° for carotid anthropomorphic vascular phantom experiments, respectively. The corresponding SPA is − 173.47 ± 0.065°, − 115.57 ± 4.83° and − 233.9 ± 8.12° for the PVA phantoms, respectively. The in vitro experimental results indicated that in the ICAs, WSS and CS were of different phases. The SPA was more negative in the ICAs as verified and indicated by our in vivo mouse experiments (− 280.08 ± 13.12°) compared with CCAs (− 141.97 ± 8.03°) and ECAs (− 170.07 ± 9.24°). The results indicate that SPA may have a key role in prediction of the atherosclerosis-prone regions, which now motivates future study on larger sample sizes for further verification.
